# Exploring the complex realities of nursing work in Kenya and how this shapes role enactment and practice—A qualitative study

**DOI:** 10.1002/nop2.1812

**Published:** 2023-05-24

**Authors:** Daniel Mbuthia, Sharon Brownie, Debra Jackson, Gerald McGivern, Mike English, David Gathara, Jacinta Nzinga

**Affiliations:** ^1^ KEMRI Wellcome Trust Research Programme Nairobi Kenya; ^2^ School of Nursing, Midwifery & Public Health University of Canberra Bruce Australia; ^3^ School of Medicine & Dentistry Griffith University, University Drive Nathan Queensland Australia; ^4^ Centre for Health & Social Practice Hamilton New Zealand; ^5^ University of Sydney Sydney New South Wales Australia; ^6^ University of Warwick Coventry UK; ^7^ Centre for Tropical Medicine and Global Health, Nuffield Department of Medicine University of Oxford Oxford UK; ^8^ London School of Hygiene and Tropical Medicine London UK

**Keywords:** care activities, care plan, coping, nurse roles, professional boundaries

## Abstract

**Aim:**

We explore how nurses navigate competing work demands in resource‐constrained settings and how this shapes the enactment of nursing roles.

**Design:**

An exploratory‐descriptive qualitative study.

**Methods:**

Using individual in‐depth interviews and small group interviews, we interviewed 47 purposively selected nurses and nurse managers. We also conducted 57 hours of non‐participant structured observations of nursing work in three public hospitals.

**Results:**

Three major themes arose: (i) Rationalization of prioritization decisions, where nurses described prioritizing technical nursing tasks over routine bedside care, coming up with their own ‘working standards’ of care and nurses informally delegating tasks to cope with work demands. (ii) Bundling of tasks describes how nurses were sometimes engaged in tasks seen to be out of their scope of work or sometimes being used to fill for other professional shortages. (iii) Pursuit of professional ideals describes how the reality of how nursing was practised was seen to be in contrast with nurses' quest for professionalism.

## 1 INTRODUCTION

There is a growing attention towards nursing roles in the contemporary healthcare setting, with debates about nursing roles and why nurses struggle to meet their professional standards (Harvey et al., 2020; Mantovan et al., 2020). The dilemmas and struggles nurses face in meeting individual patient care needs and consequent failure to meet their professional expectations of care are well documented (Gathara et al., 2020; Mbuthia et al., 2021). Nursing in resource‐constrained settings is typified by daunting working conditions characterized by high workloads to inadequate resources, high nurse‐to‐patient ratios, poor physical infrastructure and inadequate supplies (McKnight et al., 2020; Strong, 2017). This, coupled with lack of role clarity and minimal emotional support means that nurses face even more dilemmas and challenges in operationalizing professional ideals of optimal patient care (Harvey et al., 2020; Kruk et al., 2018).

Further, nurses often find themselves burdened with non‐nursing duties including administrative tasks, transporting patients and housekeeping duties which take them away from specific nursing duties (Grosso et al., 2019; Mantovan et al., 2020; Netshisaulu et al., 2019). Cumulatively, these have been shown to contribute to ‘missed care’ where fundamental aspects of nursing work are delayed or neglected as nurses grapple with competing work demands and this may result to a compromise in the quality and safety of patients (Ball et al., 2018; See et al., 2020). Relatedly, intensification of nursing work is also related to a phenomenon described as ‘busyness’—involving nursing tasks being performed in a hectic manner with the aim of task completion, leading to a task‐based nursing approach to care over knowledge‐based care (Govasli & Solvoll, 2020; Safazadeh et al., 2018) and this has been shown to limiting the provision of holistic patient care (Cho et al., 2020; Harvey et al., 2020; Nzinga et al., 2019). Despite these evidence on the practical limitations of performing nursing, the training and socialization of nurses into the profession continue to be characterized by incongruence between how nursing is taught and how it is actually applied in practice (Kerthu & Nuuyoma, 2019) and studies have shown that this theory–practice gap is problematic, not just for students but also qualified nurses (Greenway et al., 2019; Salifu et al., 2019).

### 1.1 Nursing context in Kenya

Nursing training in Kenya is offered in three entry levels; certificate, diploma and degree, as well as specializations offered in higher diploma, masters and PhD levels. To improve the status of nursing as a profession, the Nursing Council of Kenya (NCK) has been making efforts to advance nurses training in Kenya, with fewer certificate programmes being offered in favour of diploma and degree in nursing. Kenya has also been scaling up training of nurses; the NCK which regulates nursing training reports there are 121 accredited nursing/midwifery training institutions in Kenya (Nursing Council of Kenya, 2022). Paradoxically, this has also coexisted with nursing staffing shortages, especially in public hospitals, with nursing densities varying from 9.7 per 10,000 population in Nairobi to as low as 0.1 per 10,000 population in Mandera county (Ministry of Health, 2015).

Under a devolved system of government in Kenya, health was devolved to be managed at the county level. The national government allocates block grants to the county governments, who decide how these finances are appropriated (Tsofa et al., 2017). Therefore, huge disparities exist on the available human resources for health across the counties. Despite NCK efforts to come up with a nursing scope of practice and efforts to mainstream the nursing process, reduced hospital autonomy in financial management, hospital administration, procurement and human resource management functions post‐devolution, remain a challenge in implementation (Barasa et al., 2017; Wagoro & Rakuom, 2015).

The country has made several advancements aimed at transforming and professionalizing nursing, with increased investments in both expanding nursing training and developing a professional scope of practice for nurses (Nursing Council of Kenya, 2020). Additionally, to bridge theory–practice gap and to offer a scientific and systematic approach to deliver high‐quality patient‐centred care, the Kenya‐Nursing process was operationalized in public hospitals and incorporated in the training curricula for nurses, the framework for nursing care and the nurses' scheme of service (Mangare et al., 2016; Wagoro & Rakuom, 2015). While nurses have embraced the implementation of the nursing process, failures in implementation have been associated with negative attitudes and lack of support for the nursing process by senior nurse managers, chronic staffing shortages as well as financial restrictions (Lotfi et al., 2020; Mwangi et al., 2019; Rakuom et al., 2016; Wagoro & Rakuom, 2015).

Nonetheless, Kenyan nurses still struggle to provide the appropriate care and practise to the full extent of their training and education due to staffing shortages and huge workloads (English et al., 2020; Gathara et al., 2020). Further, the practice of nursing care to full capacity depends on conducive work environments where nurses have the liberty to make fundamental decisions and autonomous clinical actions (Rakuom et al., 2016). Our study therefore sought to explore how nurses navigate competing work demands in resource‐constrained settings and how this shapes the enactment of nursing roles.

## 2 METHODS

### 2.1 Study design

This was an exploratory‐descriptive qualitative research study. This design was appropriately selected as it allowed the researchers to explore and contextualize how nurses enacted their roles within resource‐constrained county referral hospitals but also provided a picture of what happens in inpatient care settings.

### 2.2 Study setting and study participants

**FIGURE 1:**
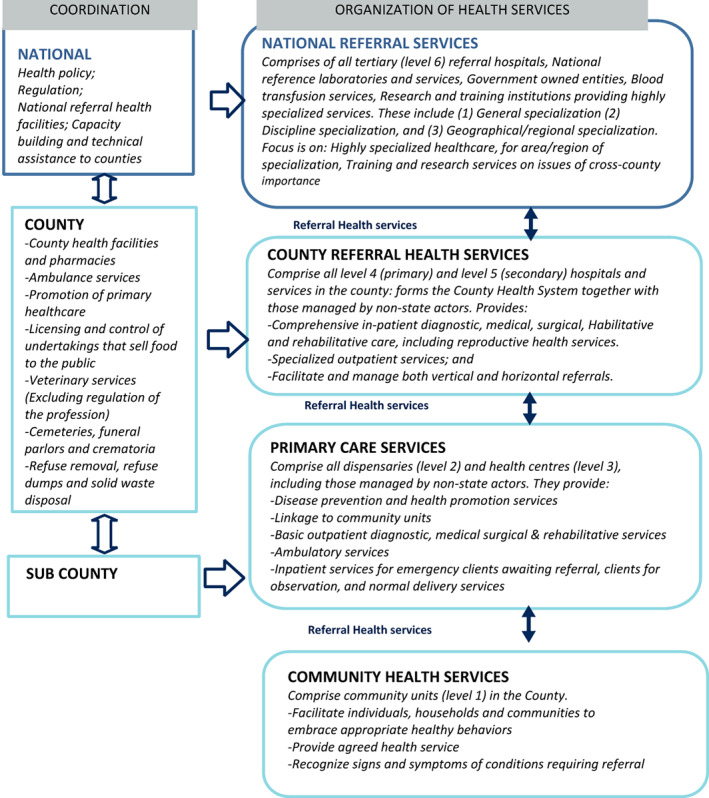
Source: Kenya Health Policy (Ministry of Health, 2014).

The Kenyan Health system is organized as illustrated in Figure 1.

The study was conducted in three purposively selected county referral hospitals offering inpatient, outpatient care and referral care according to the Kenya Essential Health Package guidelines illustrated in Figure 1. The hospitals were purposively sampled to capture variation in terms of size and bed capacity, health workforce size, universal health coverage (UHC) implementation status and geographical locations (serving different urban/peri‐urban and rural areas, Table 1).

**TABLE 1:** Below describes the selection criteria for each of the study sites.

Study site	Selection criteria
County A referral hospital	Pilot site for universal health coverage (UHC) and thus with advanced coverage of the UHC benefit package among its population (Muinde & Prince, 2022)
	Serves urban population
	Staffing: 230 Nurses
	Bed Capacity: 270 beds
County B referral hospital	Independently implementing measures to achieve UHC with notable success in access to essential health services similar to those in the UHC benefit package
	Serves a rural population.
	Staffing: 170 nurses
	Bed capacity: 400 beds
County C referral hospital	Not a pilot site for UHC
	Serves both urban and rural population given that it's adjacent to a city.
	Also, logistically convenient as near Nairobi where national level stakeholders are based and also the operational base of the researchers thus allowing collection of observational data.
	Staffing: 199 nurses
	Bed capacity: 430 beds

Participants were purposively selected based on their work experience (ranging 1‐ over 20 years' experience), different areas of specialization, gender, varying ages and professional designation based on grades, that is, nurse managers (ward in‐charges) and frontline nurses (junior nurses) and national level nurse managers to ensure diversity (see Table 2 for more details). National level nurse managers were purposively selected to offer expert advice on experience in nursing workforce planning and management, and experience in developing and implementing nursing policies in the country. A total of 47 participants participated in in‐depth interviews [IDIs] and three small group interviews of three frontline nurses each. Small group interviews were adopted to enable more detailed exchange regarding the topic, allowing multiple insights and addressing any emerging disagreements.

**TABLE 2:** Study participants.

Location	Role	Total (n = 47)	Female (n = 38)	Male (n = 9)
County A	Frontline nurses (Of whom 3 participated in small group interviews & 3 in individual interviews)	6	5	1
	Frontline nurse managers	5	5	0
	Mid‐level nurse managers	3	2	1
	Senior nurse managers	3	1	2
County B	Frontline nurses & interns (Of whom 6 participated in small group interviews & 4 in individual interviews)	10	9	1
	Frontline nurse managers	7	6	1
	Mid‐level nurse managers	2	2	0
	Senior nurse managers	4	3	1
National level	Senior nurse managers	7	5	2
	[Senior managers at Ministry of Health in charge of development and implementation of nursing guidelines and policies (3), Senior managers at Nursing Council of Kenya charged with regulatory oversight of nursing education and practice in the county (2) Nurse managers at National Nurses Association of Kenya which represents the professional and social well‐being of all nurses (2)]			

Data collection was done by DM and took place between September 2019 and September 2020. Data collection and analysis were undertaken iteratively, and the sampling process was informed by preliminary analysis as described in detail in the next section under ‘Data analysis’. The IDIs lasted between 41 min and 1 h 35 min (average of 1 h 3 min). Small group interviews lasted between 1 h 8 min and 1 h 30 min (average 1 h 20 min). Some of the questions participants were asked in the IDIs and small group interviews included: ‘Thinking about the tasks that nurses perform on a day‐to‐day basis, which ones take up most of your time? Which ones take the least amount of your time? Are there any activities or tasks you would like to get done, but cannot find the time to do? Of the tasks that nurses are mandated to perform, do you think there are some tasks that they are currently undertaking that sometimes maybe are outside their mandated roles? How would you like your work to develop in the future? What are the positive things in your work environment that can help you get there? What are some of the personal skills and personal attributes that you need to get there? What are some of the challenges you may face in getting there’?

To complement data from the IDIs and small group interviews, we conducted 57 h of non‐participant structured observation—involving observing participants without actively participating—across the three counties, but exclusively in County C as we felt we had reached point of data saturation from IDIs and group discussions from the first two hospitals. Given that data collection and analysis were undertaken iteratively, theoretical saturation was achieved before entry to hospital C. However, to capture the contextual variation, we exclusively conducted structured observations in Hospital C mainly to provide a more detailed view of emerging findings using an observation guide similar to the interview guides adopted in Hospital A & B and complemented by informal conversations with the nurses. We observed the nurses and the managers in their natural setting as they conducted various nursing tasks ranging from routine provision of care, ward rounds, handovers, ward meetings and during social interactions, for example, tea breaks.

The events observed were recorded as field notes and the informal conversations were used to reflect on ongoing analysis. Overall, we conducted a total of 57 h of non‐participant observation, with 10 h of non‐participant observations in county A, 8 h of non‐participant observations in county B and 39 h of non‐participant observation in county C.

### 2.3 Data analysis

DM & JN developed interview summaries after each interview to familiarize themselves with the emerging data. The audio data were then transcribed verbatim and together with the field notes imported into NVIVO 12 qualitative software for data management. The data were then inductively coded adopting aspects of grounded theory approach which encompassed concurrent data collection and analysis (Corbin & Strauss, 1990). Open codes from the initial coding were compared and then grouped into themes in an iterative process involving data collection and analysis. The emergent themes were used to revise data collection tools for subsequent data collection, and in making comparison between the emergent themes and the new data as described by Corbin & Strauss (1990). These comparisons informed the second level of coding which involved developing more interpretive higher‐level themes by grouping together smaller themes into bigger themes and comparing the relationship between the themes in a process of selective coding in order to explain and make sense of the phenomenon under study. For instance, minor themes like prioritizing tasks, missed care, triaging patients and delegating tasks were all grouped under rationalization of prioritization decisions.

### 2.4 Trustworthiness

Several techniques were used to ensure trustworthiness by implementing a criterion of measures for ensuring credibility, dependability, confirmability and transferability (Connelly, 2016). Credibility was achieved by using more than one method of data collection. Additionally, two researchers debriefed after the interviews and developed interview summaries. Two researchers independently coded the initial interview data and agreed on a coding framework, from which emergent themes and categories were developed. Subthemes, themes and categories were reviewed and discussed during research team meetings and disagreements discussed until consensus reached Confirmability was achieved by ensuring that the results are supported by direct quotes from participants. By outlining a step‐by‐step procedure for data collection and analysis, the researchers were able to establish dependability and make their work replicable. Transferability was ensured by sampling participants with diverse characteristics.

## 3 RESULTS

### 3.1 Study participants

A total of 47 nurses participated in this study. The table below shows the distribution of the study participants.

We present the results of this study under in three thematic areas (‘Rationalization of prioritization decisions’, ‘Bundling of tasks’ and ‘Pursuit of professional ideals’) as depicted in Table 3.

**TABLE 3:** The data structure.

Thematic codes & concepts	First‐order subthemes and codes	Illustrative evidence provided under each subtheme
Rationalization of prioritization decisions	*Missed care*	*Nursing tasks such as observations, wound care being missed or postponed*. *Ward rounds often skipped due to nursing shortage and nurses prioritizing nursing care tasks*. *Challenges with proving patient education due to workload*.
	*Prioritization of tasks*	*Prioritization of biomedical interventions and technical nursing tasks,* i.e. *giving medication, documentation, taking patients for investigations*. *Prioritization of acute conditions, emergencies or surgical patients who need close monitoring*. *As such tasks related to patients' personal needs, counselling, health education often not done*. *Other patients (stable) feeling neglected*.
	*Triaging patients*	*Care for critically ill patients (category A) coming first*. *Nursing handovers not covering all patients; focusing on acutely ill patients who need close monitoring,* i.e. *diabetic patients*. *Delegating care for stable patients to relatives/ students as nurses focus on*.
	*Delegating tasks*	*Assigning relatives/ students/ casuals to support with some of the nursing tasks*. *Mothers doing most of the feeding in NBUs (including NG tube feeding)*.
	*Coming up with a ‘workable standards’ of care*	*Nurses only providing the basic care that they must accomplish within their shifts*. *Providing the minimum standards of care*. *Nurses coming up with own standards of care,* i.e. *adjusting observations’ frequency to match what nurses are capable of achieving*.
	*Using students to fill the gap*	*Using students to fill in the nursing shortages*. *Pairing new students to learn from more experienced students*. *Consequently, challenges in nurses training and transferring skills to students*. *Students learning negative practical norms and likely to perpetuate same practices*.
Bundling of tasks	*Performing tasks outside scope*	*Nurses performing tasks such as fixing nasal‐gastric tubes*. *Nurses doing cannulation of patients and catheterization*.
	*Nurses used as gap‐fillers*	*Nurses clerking patients to cover shortage of Clinical officers*. *Nurses providing nutritional advice in absence of nutritionists*. *Nurses testing for HIV, blood sugar, malaria*. *Nurse managers coordinating multiple roles due to attrition and slow pace in filling the vacant positions*. *Nurses performing clerical tasks*.
	*Moral dilemma and medico‐legal issues*	*Dilemmas on whether to perform tasks outside of scope for the sake of the patient*. *Fear of prosecution and blame when performing tasks outside of their scope of practice*.
Pursuit of professional ideals	*Nursing ‘professionalizing’*	*Nursing emerging as ‘its own profession’ rather than ‘doctor's assistant’*. *Nurses going back for further training and specialization*.
	*Advancements in nursing training and areas of specialization*	*Advancements in nursing training up to PhD level and multiple specialties*.
	*Role blurring*	*Blurring of decision‐making roles regarding patient care between nurses and medical officers*. *Tensions over responsibility for certain roles between nurses and medical officers (*i.e. *collecting and submitting specimens for lab tests)*.
	*Challenges in implementing the scope and practising professional ideals*	*Lack of clarity in scope of practice*. *Limited autonomy in decision making* *Care fragmented taking a routine and task‐based approach*. *Consequently, challenges in transferring knowledge into practice*.

### 3.2 Rationalization of prioritization decisions

Participants described being burdened with enormous workloads and limited resources, such that daily nursing tasks became negotiated and crafted in ways that enabled normalization of care rationing decisions. Thus, medical tasks were prioritized over physical and emotional caring tasks. The rationale here being that tasks that were time bound and related to the stability of the patient were perceived as indispensable and became prioritized over emotion works and personal care. Oftentimes, tasks ended up not being ‘missed’ but nurses made deliberate decisions on which tasks to prioritize over others, as described in the interview extract below:

“You'll just give medication [and]… fluids to these patients, you will take vitals to this patient, but that personal care … you won't even have time for giving health talks to these patients.” [Frontline Nurse_012, County A]

Technical tasks such as giving medication, dressing, documentation in the nursing Kardex (a *file nurses use for documenting patient care)*, monitoring of patients with critical conditions and taking patients for investigations were thus viewed as ‘must do tasks’ and were prioritized. While categorizing patients enabled nurses to allocate more time to the critically ill patients, participants highlighted that they still struggled to ensure that all care was done, with some of the required nursing tasks being missed or handed over to nurses covering the next shift.

“We categorize our patients… those that are very sick who cannot do anything for themselves, that is category A…. so, you first handle this. There are those who are able to do something for themselves, you tell them to…. Then there are those who have very bad wounds, you dress their wounds, the wounds that are not very bad, you do not attend them that day…. There are others who would require even observations, at times you are not able. In fact, that is another area that you don't meet the requirement because you are alone, by the time you are done giving medication… it is already 1:00 PM, you are preparing to handover” [Frontline Nurse Manager_005, County B].

As nurses strive to achieve completion of ‘core’ and ‘indispensable’ tasks, minimal time is allocated to caring works and communicating with patients thus impacting on nurse–patient relationship. Nurses described working in a hurry and using strategies to minimize contact with patients by blocking requests or questions from patients.

“Whatever you are doing, you are doing in a hurry, maybe the patient was asking you some question, you did not have time to answer, because they are wasting your time [and] you want to rush.” [Frontline Nurse Manager_009, County B]

As such, nurses also described lacking the time to fully develop and implement the nursing care plans aimed at meeting individual patient care needs.

“Planning for the patient and the planning for what services you're supposed to do for each individual patient now is no longer practical. The plan is there but no implementation, no evaluation…. that nursing diagnosis, those things we assessed… are not actual [done] as they are required.” [Mid‐level nurse_003, County A]

This discrepancy between what nurses are trained to do and how nursing is practised contributed to a sense of underachievement with some participants describing nursing as ‘decaying’ and profuse with rationing care and focusing on the indispensable tasks thus pulling nurses away from achieving professional standards.

“You give a skeleton of what you are supposed to give. Now, I am supposed to sit with a patient here, give health education so that she doesn't come back with the same problem… If she goes without information, that is a gap…. That is why I am saying nursing is decaying.” [Frontline nurse manager_004, County B]

In such work contexts replete with heavy workloads and pressures to ensure task completion, nurses described providing minimum standards of care, improvising and coming up with their own acceptable ‘working standards’ as an important coping mechanism.

“You'd like to observe that patient four times, but you can't. So, you at least observe twice. You know now that is not what is prescribed… Actually, sometimes you let them [nurses] do what they can.” [Mid‐level nurse manager_016, County A]

Nurses also described adopting informal task‐shifting strategies. This included delegating some of the tasks like turning and feeding to patients' relatives or non‐nursing casual staff (in county A) despite concerns about the quality of how these tasks were performed when not supervised by nurses.

“ …there are certain fractures you don't just need to move just anyhow, and you can't be there in each and every patient's bed so you just leave them [casuals] to do what they will do with the assistance of the relative, which is quite not appropriate, you need to be there so that you find out how the patient is being supported, how the patient is being moved, and if the right assistance was there” [Frontline Nurse manager_011, County A]

Nurses felt delegating tasks to patients' relatives dented the image of nurses, especially when relatives felt those were nursing duties. Nurses, however, rationalized this as a means of involving them in the care process, especially in circumstances when faced with overwhelming tasks.

I can see some mothers coming in to feed their babies in the critical care section of the room. The nurses were going about with treatment and documentation as the mothers continued feeding their babies through the NG [Nasogastric] tubes (while unsupervised). In the stable room section, the mothers have also arrived, and they begin cup feeding as treatment progresses. [Observation Notes, County C]

“…and this one has a disadvantage with kind of the reputation‐ You will overhear people out there saying ‘you are asked to [NG tube] feed your baby, it's not the nurses’. They don't understand that you are doing that because you are straining, you are alone. So, we involve them in the care.” [Small Group Interview_011, County B]

These improvisations were so deeply internalized that it impacted on how students are mentored and socialized into the profession. We observed how common it was for nurses to use students to cover the wider staffing deficits. In one of the hospitals (County C), senior students were paired to work with, and train the less experienced students, without supervision and mentorship of qualified nurses.

By now some students are already in the acute room with the medicine trolley and they have begun giving treatment. I notice that they are working alone, unsupervised, as they dilute and give injections… The in charge would occasionally look over from afar to observe as the students continued working. At some point during the ward round, the in charge left and went over to supervise a student who putting a patient through an IV treatment before returning back to the ward round. The other students continue going round giving treatment while unsupervised. [Observation Notes, County C]

This raised concern among senior nurse managers about training and mentorship of students unconsciously perpetuating negative socialization into the profession of new nurses.

“Even the ones we are training may not reach that standard… they're used to seeing short cuts and many other things that are done wrongly… If a student think feeding a patient through an NG (Nasogastric) tube is a relative's procedure, then that's what they'll do and practise. So, in many years to come we will not have a nurse, we will just have quacks.” [Mid‐level nurse manager_003, County A]

In summary, we describe how nurses navigate constrained work contexts characterized by huge workloads and staffing shortage. While rationing care, informal task shifting and coming up with their own working standards enabled nurses navigate demanding workload, it also contributed to a sense of underachievement and negative socialization of students training under such work contexts.

### 3.3 Bundling of tasks

Owing to their constant physical presence in the ward, nurses were often informally delegated tasks and expected to perform roles outside of their mandate. For instance, participants described doctors expecting nurses to take specimens to the labs even when this was not their role and being expected to commit to undefined administrative works.

“You are told, ‘the doctor is discharging a patient, you come and bill the patient’. You fill those books. Here, we have I think five books you are supposed to fill. Records work! …We are doing a lot of clerical work here!” [Frontline Nurse manager_010, County A]

Additionally, nurses also highlighted the tensions and pressures in performing some of the tasks outside their mandate, especially when performance of other nursing care procedures were pegged on having these tasks done by other professionals.

“like there is this testing of blood sugars, you are in the ward, you have the gluc sticks [diabetes test strips] and you know how to do, you will be told ‘That it is supposed to be done by the lab technician.’ But you can't just sit and watch a DM [Diabetes Mellitus] patient who have changed condition…there is a wound that will require maybe vacuum dressing, they will keep on saying that this is a doctor's procedure, but you are the nurse in the ward… and you need to dress, they will keep postponing… you will do.” [Frontline Nurse manager_009, County B]

Nurses were also sometimes used as fillers to cover for the other professionals' shortages but with noted weak regulation. Often nurses were expected to undertake roles outside of their scope of practice, for example, fixing cannulas or catheters because of pressure to start patients on medication or to alleviate a patient's condition during emergencies.

For nurses, performing tasks outside their mandate reportedly led to moral dilemmas—on one hand, nurses felt obliged to perform these tasks as they would otherwise end up undone. However, they worried about the regulatory implications for performing tasks outside their scope of practice.

“It has some implications because now if I do what I am not supposed to do even if I felt that I was helping [a patient], now when it comes to legal issues I am in and again I cannot just stop and watch.” [Frontline nurse manager_008, County B].

Shortages of other healthcare professionals also means that nurses are also expected to fill those gaps.

“…at the end of the day they also spread widely to cover areas of other professionals… because I will offer nutritional advice for example, because I have some training in nutrition… You know there are many things that I can, beyond nursing and which are perceived to be other people's fields and so when you have a nurse in that renal unit for example, you are assured that even if I do not have a nutritionist my patient will be able to get nutritional advice and how they will be able to manage their salt levels, their potassium” [Senior Nurse Manager_003].

Senior nurse managers at the county level also highlighted being used to fill job functions left vacant due to attrition, with some coordinating up to three different job functions at a time. The slow pace at which these positions are filled means that these functions end up being delegated to other nurse managers at the discretion of the county governments.

“Like for example, what we are coordinating, a county at their own discretion, they can give coordination to anybody they want. For example, you see here I'm having three jobs…. You've seen it in me. You already have two retired from this office… it therefore need [necessitates] the person who's there [remaining] to take over.” [Senior nurse manager_013, County B]

In sum, we describe weak regulations over nursing roles and how nurses are often used to fill for other professional shortages and being expected to perform tasks that fall outside of their mandate resulting in moral dilemmas and fear of medico‐legal issues related to performing tasks outside their mandate.

### 3.4 Pursuit of profession ideals

In response to the challenges of practising nursing work, participants spoke on how ongoing tremendous changes in nursing practice in Kenya were helpful in seeking to establish nursing as an independent profession away from the long‐held view of nursing as doctors' handmaiden.

“People are going back to school, they are investing much in their education, and also the patient has developed that trust with a nurse, and also it has become its own profession rather being a doctor's assistant.” [Frontline Nurse_014, County B]

At the core of this were advancements in nursing education and entry standards contributing to nursing ‘professionalizing’ and acquiring status as an independent profession with its body of knowledge. These changes were associated with a calling for proficient application of knowledge in nursing practice in order to influence patient outcomes.

“We have had nursing grow from certificates and we now do have PhD holders and we also have professors as well. So, with all the change in the academic, there is also need for the same change to be felt in our hospitals” [Small group interview_004, County A]

However, the context in which nursing is practised presents great tensions with this forward‐looking quest for nursing to practise to their scope of practice and professional ideals. Thus, nurses argued for the need to clearly define nursing work and clearer articulation of the nursing process where “they use their wealth of knowledge to plan, implement and evaluate care.” [Senior Nurse Manager_007].

We observed that during ward rounds, conversations regarding patient care mainly revolved among the clinicians and the physicians. Nurses only mostly took notes on the recommendations made regarding the care of the patients, evident that nurses had little involvement in the discussion and decision making regarding the management of the patient.

The ward round begins… the main consultant probes the C.O [Clinical officer] intern and asks questions about the patients' condition… More questions follow, directed to the C.O interns …All this while the nurse is not asked a single question about the patient's condition. The nurse is again asked to take the BP [Blood pressure] of the next patient and the physician goes on to lecture the interns about the patient's condition… Occasionally the physicians consult… The nurse on this ward round is only consulted when a BP chart or a vitals chart is needed or when the physician needs gloves to check the patients. [Observation Notes, County A]

More broadly, participants highlighted the need to expand the autonomy of nurses in decision making regarding patient care, such that nursing can move beyond the traditional nurse–doctor power hierarchy and nurses can have more latitude in decision making regarding patient care through implementing the nursing process without necessarily having to wait for doctors' orders.

“… they [doctors] are prescribing drugs, ‘vital signs observations six hours’, and you see that will remain there [in the patient file] until when the patient is even being discharged… is it making any logic?” [Senior Nurse Manager_007].

Participants also described this as the shortcoming of the nursing profession‐ that nursing remains task oriented, failing to exert its body of knowledge in practice, describing this as the weakest link, especially when care is not planned and evaluated.

“…that is the weakest point in nursing, most of them just work on impulse, they don't plan for care, they implement orders, most of them you'll hear ‘The doctor has said we should do this.’ They don't even stop to think specifically, why am I doing this? Why paracetamol and not aspirin for example… Now that is the science that we want nurses to go into, that any form of care that they are offering, they must have a scientific rationale and at that request, further knowledge and planning” [Senior Nurse Manager_007]

“You learn so much pharmacology, you learn so much immunology and patient's procedures, but now when you come to the ward you are not even able‐ if you are asked is this dose the correct one for this patient as per their age, weight and all that? The truth is no because you do not have that time to ensure that the dosage is correct. You just give because someone else prescribed. You see? You are not able to sit down and plan a daily care plan for a patient depending on their needs” [Small group interview_010, County B]

At the system level, senior nurse managers highlighted challenges in regulating and enforcing nursing standards especially after devolution. This was attributed to the nursing council lacking enforcement capacity over county governments.

“We do conduct what we call support supervision… the planning for the visits and the feedback involves the CEC [Chief Executive Committee Member] of that county in charge of health as well as nursing. I know it is a challenge… the much I would say we are able to do‐ because in terms of implementation, that has a lot of weight on the county…it has become a challenge after devolution.” [Senior Nurse Manager_001]

In sum, we describe the advancements in nursing profession in Kenya, highlighting a move towards ‘professionalization’ in nursing. We also highlight the tensions between the forward‐looking direction that nursing aspires to in practising their ideals versus how nursing is practised in reality.

## 4 DISCUSSION

Evidence has shown that nurses have multiple roles in contemporary healthcare settings, fraught by nursing staffing deficits and other systemic constraints, a situation that ultimately shapes how nursing roles are constructed and enacted (McKnight et al., 2020; Nzinga et al., 2019). We explored how nurses navigate such competing work demands in resource‐constrained settings and how this shaped enactment of nursing roles.

Our findings show that in coping with demanding workload, nurses reconstructed their work, prioritizing technical roles over caring and emotional supportive roles thus cementing how nursing roles were perceived and enacted. The standardization and routinization of care while ensuring efficiency, removed nurses from the individualized model of care thereby creating an image of the nurse as both emotionally distant and detached in care by the patients.

To cope with the dilemmas on how to allocate their time amidst competing work demands, coping mechanisms employed by nurses had detrimental effect not only to care delivery but to professional identity and job satisfaction. For instance, nurses rationalized giving much priority to technical biomedical interventions, that is, initiating treatment, over caring works as these were thought to be dedicated by time and could not be safely delegated to patient care givers. These findings corroborate with other study findings which show that when work demands exceed nurses' capacity to get the job done, fundamental nursing care especially related to patient's personal or non‐clinical needs is often neglected in favour of technical tasks (Nzinga et al., 2019; Richards & Borglin, 2019). As such, nurses in our study adopted strategies such as deterring services by blocking interruptions or requests from patients, which allowed them to manage competing work demands and priorities and thus giving them a sense of control over their work. Elsewhere, nurses have also been shown to adopt strategies such as reducing the quality of planned nursing care so as to manage their time and stick to their routine (Mantovan et al., 2020) and this may ultimately contribute to depersonalization and role incongruity (Harvey et al., 2020).

Managing competing work demands forced nurses in our study to engage in inappropriate task shifting. Nurses used relatives or nursing students as sources of support and sometimes assigned them nursing and caring tasks such as giving medication and doing observations, which in turn allowed them time to concentrate on other patients or more technical tasks (Fitzgerald et al., 2020;McKnight et al., 2020; Nzinga et al., 2019). This however raised concerns about patient safety, the image and confidence of the profession and reinforced normalization of these practical norms which negatively impacted on the mentorship and socialization of students. These pragmatic micro‐choices of organizing care within demanding workloads consequently become the new norm of how care is delivered, sometimes in contravention of official care guidelines (Mbuthia et al., 2021; Olivier de Sardan et al., 2017).

Further, strategies adopted to cope with work demands included intensification of work, with priorities often being set towards task completion. This undermined nurses' capacity to plan and evaluate nursing care plans as idealized and further limited time dedicated to train and mentor students (Foolchand & Maritz, 2020). While delegating tasks to students and patients' relatives ensured productivity, in the long term, this may undermine application of nursing expertise in care, as care follows a particular routine and thus becomes task‐based, with care taking priority over student education. Studies have shown that routine‐based approach to care limits students' application of theoretical knowledge in practice as they tend to emulate how experienced nurses practise, contributing to theory–practice gap (Safazadeh et al., 2018).

While the nursing process provides for a systematic and rational method for nurses to plan and provide individualized care, in practice, nurses were unable to fully execute the nursing process. Our work revealed that the implementation of task‐oriented nursing activities took precedence, thus undermining other critical steps (assessment, diagnosis, planning and evaluation) in the care process where the knowledge and expertise of nurses are needed even more. Without proper guidelines, decisions to prioritize and ration care remain largely at the nurses' intuition and discretion rather than through analytic or logic processes (Mantovan et al., 2020; McKnight et al., 2020). This is potentially dangerous and discriminatory to patients as well as morally burdensome to nurses (Scott et al., 2019). Our findings illustrate how failure to meet these professional standards resulted in nurses creating their own implicit ‘working standards’. These standards consequently shaped both the identity of the nursing care profession and how nursing care is practically delivered. Furthermore, unregulated task shifting as a coping mechanism for health worker shortages often took nurses away from their ‘core’ nursing tasks (McKnight et al., 2020; Nzinga et al., 2019), but also contributed to role incongruence, moral dilemmas and fears for legal implications of practising outside their scope of practice (Mathibe‐Neke & Mashego, 2022).

With nursing in Kenya undergoing professionalization and seeking to establish itself as an independent vocation, the challenges in transferring knowledge as taught in nursing education and the bridging practice gap remains (Rakuom et al., 2016). To address some of these challenges, advancements like the mainstreaming of the Kenya nursing process and revision of the nursing scope of practice remain constrained by limited financing, lack of support from senior nurse management and poor work environment (Nursing Council of Kenya, 2020; Rakuom et al., 2016; Wagoro & Rakuom, 2015). Consequently, nursing practice remains routine based (Nzinga et al., 2019) and this undermines nurses' application of their expertise and evidence‐based practice into everyday nursing practice and limits the transfer of this expertise to nursing students.

To bridge the knowledge–practice gap, there is need for change in culture where the nursing process is not only taught in the classroom settings, but also in the clinical areas, and should serve as the benchmark for evaluating nursing practice (Rakuom et al., 2016). We recommend the development of explicit nursing standards and care guidelines that are context specific to guide nursing practice and to serve as the basis for audits and quality evaluation. Such standards should not only serve to improve professionalism, but also serve as the basis for informing decisions on staffing levels. Further, at the policy level, urgent action is needed to address nursing staffing deficits and the implications on care rationing, patient safety, quality of care and theory–practice gap, hence urgent need for increased investment in nursing staffing. At the micro level, especially in settings faced with severe shortage of nursing staff, there is need for supporting guidelines and explicit principles to aid nurses with care rationing decisions rather than care rationing being left to nurses' intuitions, decision making skills, personal feelings, or beliefs. Nurse managers also play a critical role in implementing necessary reforms geared towards quality improvements and thus have a duty to inculcate a culture of holistic care provision and caring attitude as well as training nurses on the nursing process.

### 4.1 Limitations

Our study was conducted in only three county hospitals in Kenya; therefore, the results of this study may not be generalizable to the rest of the population. Additionally, since the study was conducted in county referral hospitals, the experiences of nurses working in these contexts may be more extreme than those working in well‐resourced national hospitals or even lower‐level county hospitals.

## 5 CONCLUSION

Our findings highlight how nurses construct their roles in constrained work contexts by focusing on the must do technical tasks over bedside caring roles, rationing care and coming up with their own ‘working standards’ of care in a bid to ensure continuity of care.

We argue that without improving the working conditions of nurses and ensuring adequate staffing, nurses struggle to bring in their wealth of knowledge into practice, hence nursing work remains constructed around routines and ensuring task completion thus creating tensions with the forward‐looking direction that the profession is pursing. To implement their professional expertise, there is urgent need to cultivate organization cultures where evidence‐based nursing is supported, professional identities respected and collaborative multi‐disciplinary working facilitated.

## CONFLICT OF INTEREST STATEMENT

The authors declare that they have no conflicts of interest.

## ETHICS STATEMENT

This study was approved by Kenya Medical Research Institute ethics review committee (Approval NO: KEMRI/RES/7/3/1) and written informed consent was obtained from all participants.

## Data Availability

The data that support the findings of this study are available on request from the corresponding author through KEMRI Data Governance Committee. The data are not publicly available due to privacy or ethical restrictions.
